# Severe sepsis in women with group B *Streptococcus* in pregnancy: an exploratory UK national case–control study

**DOI:** 10.1136/bmjopen-2015-007976

**Published:** 2015-10-08

**Authors:** Asli Kalin, Colleen Acosta, Jennifer J Kurinczuk, Peter Brocklehurst, Marian Knight

**Affiliations:** 1 National Perinatal Epidemiology Unit (NPEU), University of Oxford, Oxford, UK; 2 Institute for Women's Health, University College London, London, UK

**Keywords:** PUBLIC HEALTH

## Abstract

**Objective:**

To estimate the incidence of severe maternal sepsis due to group B *Streptococcus* (GBS) in the UK, and to investigate the associated outcomes for mother and infant.

**Design:**

National case–control study.

**Setting:**

All UK consultant-led maternity units.

**Participants:**

30 women with confirmed or suspected severe GBS sepsis, and 757 control women.

**Main outcome measures:**

Disease incidence, additional maternal morbidity, critical care admission, length of stay, infant infection, mortality.

**Results:**

The incidences of confirmed and presumed severe maternal GBS sepsis were 1.00 and 2.75 per 100 000 maternities, respectively, giving an overall incidence of 3.75 per 100 000. Compared with controls, severe GBS sepsis was associated with higher odds of additional maternal morbidity (OR 12.35, 95% CI 3.96 to 35.0), requiring level 2 (OR 39.3, 95% CI 16.0 to 99.3) or level 3 (OR 182, 95% CI 21.0 to 8701) care and longer hospital stay (median stay in cases and controls was 7 days (range 3–29 days) and 2 days (range 0–16 days), respectively, p<0.001). None of the women died. Severe maternal GBS sepsis was associated with higher odds of infant sepsis (OR 32.7, 95% CI 8.99 to 119.0); 79% of infants, however, did not develop sepsis. There were no associated stillbirths or neonatal deaths.

**Conclusions:**

Severe maternal GBS sepsis is a rare occurrence in the UK. It is associated with adverse maternal and neonatal outcomes.

**Strengths and limitations of this study**

- This study collected national data on presumed or confirmed severe maternal group B *Streptococcus* (GBS) sepsis over 12 months.
- The study was thus able to robustly estimate the incidence of presumed and confirmed severe maternal GBS sepsis, and the outcomes for mothers and infants.
- The rarity of the condition severely limited the power of this study to detect differences, and thus the analyses of risk factors can only be regarded as exploratory.
- The study included both confirmed and presumed cases of severe maternal GBS sepsis. It is possible that some of the women with presumed GBS sepsis had other infecting organisms and thus, may be regarded as false positives.
- The study did not cross-check case identification with laboratory sources; therefore, estimates of incidence should be regarded as minimum estimates.

## Introduction

Group B *Streptococcus* (GBS) or *Streptococcus agalactiae* is a Gram-positive coccus found in 20% of healthy women as part of normal gastrointestinal and genital tract flora.^1^ ^2^ It is associated with pathogenicity in immunocompromised, elderly and pregnant adults as well as infants and neonates.^3^ It is the leading cause of culture-confirmed neonatal sepsis in the UK,^4^ accounting for over 50% of culture-confirmed cases that occur in the first 48 h after birth.^5^

Although sepsis-related maternal mortality remains low in high-income countries and has followed a steady decline over the past century, its incidence has increased in the UK where it is now one of the leading causes of maternal death.^6^ While it is estimated that 2.1% of maternal deaths in high-income countries result from maternal genital tract sepsis,^7^ in the UK, sepsis-related maternal deaths from all causes now represent almost a quarter of all deaths.^8^ This increase has been accompanied by a parallel rise in the incidence and severity of sepsis in the general population in Europe^9^ ^10^ and the USA,^11^ as well as an increase in maternal risk factors for sepsis such as caesarean section and obesity.^12^

Although approximately 20% of women are GBS carriers,^1^ and GBS carriage is associated with early onset GBS sepsis in neonates, only approximately 0.3% of infants of carrier mothers develop sepsis, and 3 in 10 000 die.^3^ There is no clear association with other neonatal outcomes, such as preterm birth.^13^ Antenatal GBS screening in pregnancy is thus not recommended by the UK National Screening Committee. While much research has focused on the risks and outcomes of neonatal GBS sepsis, there have been no comprehensive studies documenting the incidence and epidemiological characteristics of maternal GBS sepsis in the UK. Such data are important for assessing the burden of disease associated with GBS, and for providing guidance for the clinical and cost-benefit assessment of antenatal screening, treatment and vaccination.

The aim of this study was to use data from a larger population-based study of maternal sepsis to estimate the incidence of severe maternal sepsis due to presumed or confirmed GBS in the UK, and to investigate the associated outcomes of severe maternal GBS sepsis for mother and infant.

## Methods

### Study design and setting

A national, population-based study was conducted between June 2011 and May 2012 using the UK Obstetric Surveillance System (UKOSS) to identify all cases of severe maternal sepsis in the UK over a 1-year period.^14^ This secondary analysis identified all cases of severe GBS sepsis from the data set of women with severe maternal sepsis from all causes.

### Participants

Cases were all women in the UK identified as having severe maternal GBS sepsis. Severe sepsis was defined as described in box 1. Severe maternal GBS sepsis was defined as any case of severe sepsis with laboratory confirmation of GBS (on blood, urine or vaginal swab cultures). A ‘confirmed’ severe GBS sepsis case was defined as GBS isolated from a sterile body site, such as blood, and a ‘presumed’ case was defined as severe sepsis occurring in the context of positive GBS cultures from a non-sterile site, such as urine or vaginal swab cultures, where there was no other causal organism for the sepsis clearly identified and where the clinical staff caring for the women considered this to be the most likely cause of her sepsis. ‘Presumed’ cases were included because other studies of severe sepsis have indicated that a single causative organism from sterile site culture cannot be identified in almost 50% of cases.^15^

Box 1 **Definition of severe maternal sepsis**

Any pregnant or recently pregnant woman (up to 6 weeks postpartum) diagnosed with severe sepsis (irrespective of the source of infection). Women were not reported if they were not considered by their attending obstetrician to have severe sepsis. As a guide, a severe sepsis case would be expected to include women in one of the following groups:

1. Death related to infection or suspected infection
2. Any women requiring level 2 or 3 critical care (or obstetric high dependency unit type care) with severe sepsis or suspected sepsis
3. Clinical diagnosis of sepsis in association with two or more of the following:
  - Temperature >38°C or <36°C 4 h apart, on two occasions
  - Heart rate >100 bpm persisting for over 4 h, on two occasions
  - Respiratory rate >20/min for over 4 h or PaCO_2_ <32 mm  Hg, on two occasions
  - White cell count >17 000 or <4000/mL or with >10% immature band forms, on two occasions.

The control women were all controls identified in the study of all-cause maternal sepsis.^14^ For the purposes of this larger study, the controls were the two women who did not have severe maternal sepsis and delivered immediately before each case in the same hospital. Data from all the control women in the data set were used for comparison in this analysis in order to maximise the study power; the controls used in this analysis are thus not limited to the two women who did not have severe maternal sepsis and delivered immediately before each GBS case in the same hospital. Identical data were collected about cases and controls except for details of the sepsis itself.

### Statistical methods

The incidence of severe maternal GBS sepsis was calculated using denominator data from national birth data from 2011 as a proxy for June to December 2011 and January to May 2012; the total number of maternities (women delivering) during the 1-year study period was estimated as 799 003.^16–18^

Baseline characteristics as well as maternal and infant outcomes were compared in women with severe GBS sepsis and controls by using the χ^2^ test for association, Fisher’s exact test or the Wilcoxon rank-sum test, as appropriate. Unconditional logistic regression analysis was used to estimate ORs for binary outcomes and calculate 95% CIs. Unconditional exact logistic regression was used when appropriate. Unconditional logistic regression was also used in models comparing infant birth weight in cases and controls adjusting for the a priori confounder of gestational age at delivery. Models looking at infant outcome included a specification that the calculated SEs allow for within group correlation in order to allow for the non-independence of infants from multiple pregnancies.^19^

Stata V.11 software was used for all statistical analyses.

For exposures of 20% prevalence in the controls, with 30 cases and 757 controls, the study had 65% power to detect as statistically significant an OR of 2.0 or greater; for exposures of 2% prevalence, the power was only 9%.

This secondary analysis did not require Research Ethics Committee approval. The original study was approved by the London Research Ethics Committee (ref 10/H0717/20).

## Results

**Figure 1:**
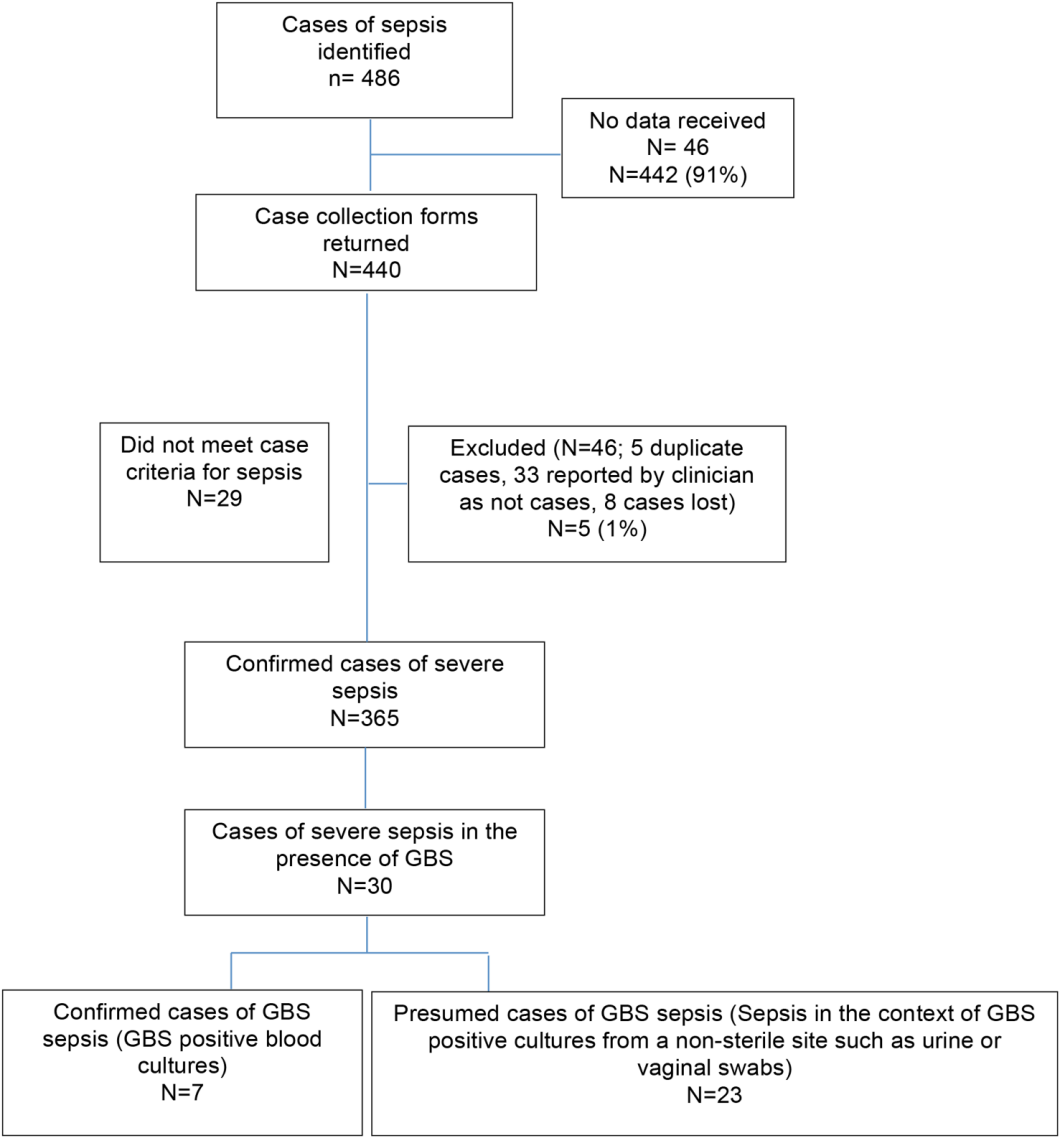
Case reporting and identification (GBS, group B *Streptococcus*).

All 214 hospitals in the UK with consultant-led maternity units participated in the study (100%). Data collection and case identification are described in figure 1.

A total of 30 women with GBS-positive cultures in the context of severe maternal sepsis were identified between June 2011 and May 2012, including 1 woman with a twin pregnancy. Of these, 7 were confirmed cases of severe maternal GBS sepsis and 23 were presumed cases of severe maternal GBS sepsis. The different sources from which GBS was isolated are listed in table 1.

**Table 1:** Culture specimen collection sites

Culture specimen collection site	Number of specimens	Per cent
Blood	7	23
Urine	3	10
High vaginal swab	11	36
Abdominal wound	2	7
Placental swab	5	17
Low vaginal swab	2	7

The incidences of confirmed and presumed severe maternal GBS sepsis were 1.00 and 2.75 per 100 000 maternities, respectively, giving an overall incidence of 3.75 per 100 000 maternities.

Maternal characteristics are presented in table 2. There were no statistically significant differences between cases and controls; however, the limited power of this analysis must be noted.

**Table 2:** Maternal characteristics

	Number (%)* of cases	Number (%)* of controls	OR (95% CI)
	n=30	n=757	
*Maternal factors*			
Maternal age (years)			
<35	24 (80)	596 (79)	1
35+	6 (20)	160 (21)	1.09 (0.46 to 2.57)
Ethnic group			
White	21 (70)	598 (79)	1
Non-white	9 (30)	156 (21)	1.83 (0.84 to 3.96)
Socioeconomic status			
Managerial	8 (27)	189 (26)	1
Other employed/unemployed	22 (73)	548 (74)	0.99 (0.44 to 2.25)
Marital status			
Married/cohabiting	25 (83)	630 (84)	1
Single	5 (17)	124 (16)	0.98 (0.37 to 2.59)
Smoking status during pregnancy			
Did not smoke	26 (87)	581 (77)	1
Smoked	4 (13)	173 (23)	0.52 (0.18 to 1.50)
Parity			
0	12 (40)	330 (44)	1
1+	18 (60)	427 (56)	1.16 (0.55 to 2.44)
Body mass index			
Normal or overweight (<30)	18 (64)	564 (77)	1
Obese (≥30)	10 (36)	170 (23)	1.84 (0.83 to 4.07)
Multiple pregnancy			
Yes	1 (3)	8 (1)	1
No	29 (97)	746 (99)	3.21 (0.07 to 25.31)
Pre-existing medical conditions			
Yes	11 (37)	242 (32)	1.22 (0.57 to 2.60)
No	19 (63)	510 (68)	1
Previous pregnancy complications			
Yes	8 (27)	148 (20)	1.49 (0.65 to 3.42)
No	22 (73)	607 (80)	1

*Percentage of individuals with complete data.

Seventeen per cent (n=5) of the cases had been prescribed antibiotics in the 2 weeks prior to delivery compared with 4% of controls (OR 4.97, 95% CI 1.38 to 14.6, p=0.015). Of the former, two had received antibiotics for fever during labour, two had been treated for a urinary tract infection (UTI) prior to delivery, and one had been treated prophylactically after spontaneous rupture of membranes given her past obstetric history of GBS in her three previous pregnancies. No other women had been given prophylactic antibiotics for GBS carriage.

There were no significant differences in the proportion of women who underwent invasive antenatal procedures (such as amniocentesis, use of a urethral catheter, intravenous or intra-arterial lines) among cases and controls (data not shown), but again the limited power of this analysis must be noted.

### Diagnosis of severe sepsis

Two women had a miscarriage; both women were diagnosed with presumed severe GBS sepsis prior to their miscarriage. The 28 remaining women had live births. Thirteen had an antenatal and 15 a postnatal diagnosis of severe GBS sepsis.

Among the 13 antenatal cases of severe sepsis in women with ongoing pregnancies, the median time between the diagnosis of sepsis and delivery was 1 h 46 min (range 1 h 3 min–36 days). Only two cases of severe antenatal sepsis were diagnosed before membrane rupture; both had clear risk factors for severe sepsis, including one case with a GBS-positive UTI and reported subsequent chorioamnionitis, and the second with pyelonephritis. Nine out of the 13 antenatal cases laboured; 7 were diagnosed with severe sepsis after the onset of labour and 2 were diagnosed with severe sepsis before the onset of labour. The four women who did not go into labour were delivered by caesarean section.

For the 15 cases of postnatal sepsis, the median time between delivery and diagnosis of severe sepsis was 41 h 45 min (range 2 h 45 min–14 days); the median time between rupture of membranes and diagnosis of severe sepsis was 2 days 13 h (range 19 h–15 days). Only one case of severe postnatal sepsis did not go into labour (delivered by caesarean section in the presence of ruptured membranes).

### Maternal delivery and outcomes

The mode of delivery for control women and the 28 women with ongoing pregnancies is described in table 3. All women delivered by caesarean section were given prophylactic antibiotics. Antenatal severe GBS sepsis was associated with raised odds of delivery by caesarean section (OR 14.1, 95% CI 3.04 to 131.9). In the 11 cases of caesarean section in the context of antenatal severe maternal GBS sepsis, indications for caesarean section included maternal compromise due to sepsis (n=9), fetal tachycardia (n=4) and failure to progress in labour (n=4). Postnatal severe GBS sepsis was associated with raised odds of instrumental vaginal delivery (OR 4.40, 95% CI 1.26 to 14.2), and decreased odds of normal vaginal delivery (OR 0.17, 95% CI 0.03 to 0.66). In the six cases of caesarean section in the context of postnatal severe maternal GBS sepsis, indications for caesarean section included breech presentation (n=2), chorioamnionitis (n=1), pre-eclampsia and prolonged preterm rupture of membranes (n=1), and failure to progress in labour (n=3).

**Table 3:** Mode of delivery and induction in cases and controls, and in relation to timing of diagnosis of sepsis

	Number (%)* of all cases	Number (%)* of controls	OR (95% CI)	Number (%)* of antenatal cases	OR (95% CI)	Number (%)* of postnatal cases	OR (95% CI)
	n=28	n=757		n=13		n=15	
Normal vaginal delivery							
Yes	5 (18)	444 (59)	0.15 (0.057 to 0.40)	2 (15)	0.13 (0.014 to 0.59)	3 (20)	0.17 (0.031 to 0.66)
No	23 (82)	310 (41)	1	11 (85)	1	12 (80)	1
Instrumental delivery							
Yes	6 (21)	99 (13)	1.80 (0.71 to 4.56)	0 (0)	0.36 (0.33 to 2.21)	6 (40)	4.40 (1.26 to 14.18)
No	22 (79)	655 (87)	1	13 (100)	1	9 (60)	1
Caesarean delivery							
Yes	17 (61)	212 (28)	3.97 (1.83 to 8.62)	11 (85)	14.09 (3.04 to 131.85)	6 (40)	1.71 (0.50 to 5.46)
No	11 (39)	545 (72)	1	2 (15)	1	9 (60)	1
Induction							
Yes	8 (30)	218 (29)	1.04 (0.45 to 2.41)	5 (42)	1.77 (0.55 to 5.62)	3 (20)	0.62 (0.17 to 2.21)
No	19 (70)	539 (71)	1	7 (58)	1	12 (80)	1

*Percentage of individuals with complete data.

There was no statistically significant difference in the number of vaginal examinations between cases and controls; the median number of examinations in both cases and controls was 3 (ranges 1–12 and 0–10, respectively).

Twenty-three per cent of women with severe GBS sepsis (n=7) had additional major maternal morbidity (OR 12.4, 95% CI 3.96 to 35.0, p≤0.001). These included pulmonary oedema (n=1), coagulopathy (n=1), postpartum haemorrhage (n=3), retroperitoneal haematoma/pseudoaneurysm (n=1), thrombocytopaenia (n=1), bilateral iliopsoas abscesses (n=1) and necrotising fasciitis (n=1). No women died in either group.

Women with severe maternal GBS sepsis had significantly higher odds of receiving intensive care compared with controls, as well as a significantly longer length of hospital stay following delivery (table 4). The median length of stay in hospital after diagnosis of severe sepsis was 6 days (range 2–39 days). Five women with severe GBS sepsis had no signs of infection during their initial hospital admission and were discharged home before being admitted with a diagnosis of severe sepsis.

**Table 4:** Maternal outcomes in cases and controls

	Number (%)* of cases	Number (%)* of controls	OR (95% CI)
	n=30	n=757	
*Maternal outcome*			
Readmission after delivery*			
Yes	7 (23)	17 (2)	13.12 (4.16 to 37.56)
No	23 (77)	740 (98)	1
Level 2 care			
Yes	17 (57)	24 (3)	39.25 (15.99 to 99.31)
No	13 (43)	733 (97)	1
Level 3 care			
Yes	6 (20)	1 (0)	182 (20.98 to 8701.16)
No	24 (80)	756 (100)	1
Major maternal morbidity			
Yes	7 (23)	18 (2)	12.35 (3.96 to 35.01)
No	23 (77)	737 (98)	1
Length of hospital stay following delivery; median (range) (days)	7 (3–29)	2 (0–16)	p<0.001

*Percentage of individuals with complete data.

### Infant outcomes

There were 29 live births to 28 women with severe GBS sepsis and no stillbirths or neonatal deaths. Cases were more likely to deliver preterm with 31% (OR 6.00, 95% CI 2.45 to 14.7) and 14% (OR 13.4, 95% CI 3.11 to 57.3) of infants being born before 37 and 32 completed weeks, respectively (table 5). Of the 9 infants born preterm, 4 (44%) were iatrogenic preterm deliveries (3 of 4 deliveries before 32 weeks).

**Table 5:** Infant outcomes*

	Number (%)† of cases	Number (%)† of controls	OR (95% CI)	Number (%)† of antenatal cases	OR (95% CI)	Number (%)† of postnatal cases	OR (95% CI)
	n=29	n=765		n=14		n=15	
*Infant outcomes*							
Neonatal unit admission							
Yes	13 (45)	60 (8)	9.48 (4.22 to 21.28)	7 (50)	11.67 (3.69 to 36.93)	6 (40)	7.78 (2.67 to 22.63)
No	16 (55)	700 (92)	1	7 (50)	1	9 (60)	1
Infant septic							
Yes	6 (21)	6 (1)	32.70 (8.99 to 118.97)	4 (29)	50.13 (10.40 to 241.62)	2 (13)	19.28 (3.55 to 104.79)
No	23 (79)	752 (99)	1	10 (71)	1	13 (87)	1
Premature birth (<37 weeks)							
Yes	9 (31)	53 (7)	6.00 (2.45 to 14.69)	4 (29)	5.34 (1.34 to 21.28)	5 (33)	6.67 (2.19 to 20.31)
No	20 (69)	707 (93)	1	10 (71)	1	10 (67)	1
Very preterm birth (<32 weeks)							
Yes	4 (14)	9 (1)	13.35 (3.11 to 57.34)	4 (29)	33.38 (7.20 to 154.84)	0 (0)	NC
No	25 (86)	751 (99)	1	10 (71)	1	15 (100)	

*Outcomes of 29 infants born to 28 mothers excluding the 2 women who miscarried.

†Percentage of individuals with complete data.

NC, not calculated due to zero cells.

The odds of the infant developing sepsis were also significantly higher in severe maternal GBS sepsis cases (OR 32.7, 95% CI 8.99 to 119.0, p<0.001); 21% of infants of mothers with severe GBS sepsis developed sepsis. Information on the causative organisms of infant sepsis was not available. All four infants of cases who were born ‘very preterm’ (<32 weeks gestation) were born to women with antenatal sepsis; three were delivered electively by prelabour caesarean section, two in the presence of ruptured membranes and one in the presence of intact membranes, and one was delivered spontaneously vaginally.

## Discussion

### Main findings

This study suggests that severe maternal GBS sepsis is rare in the UK, with an incidence of confirmed severe maternal GBS sepsis of 1 case per 100 000 maternities. Severe maternal GBS sepsis was associated with additional maternal morbidity, longer hospital stays and increased odds of maternal readmission compared with controls. It was also associated with increased odds of infant sepsis and a longer infant stay in neonatal special care units. There were no maternal or neonatal deaths or stillbirths associated with severe maternal GBS sepsis during the course of the study; however, given the small number of cases, this should be interpreted with caution.

### Strengths and limitations

Despite having collected national data over 12 months, the rarity of the condition severely limited the power of this study to detect differences and thus, it can only be regarded as exploratory. It is possible that real differences in maternal characteristics, such as non-white ethnicity, obesity and multiple pregnancy, have been subject to type II error and we were unable to identify these as statistically significantly associated with maternal severe GBS sepsis. Thus, although real differences might exist, we did not identify any statistically significant risk factors. We included both confirmed and presumed cases of severe maternal GBS sepsis since in the previous studies almost 50% of cases of severe sepsis had no definitive causal organism identified from sterile site culture.^15^ UK national guidelines recommend that blood cultures are performed in all women meeting our definition of severe sepsis,^20^ ^21^ but from these data we do not know in what proportion of women this was undertaken. It is possible that some of the women with presumed severe GBS sepsis had other infecting organisms and may be regarded as false positives. UKOSS is only able to collect anonymous information; we were thus not able to cross-check case ascertainment with national laboratory or other data, which may have identified additional cases. However, cross-checking with national data on maternal deaths showed that there were no maternal deaths from GBS sepsis during this period;^8^ it is thus reassuring that this study did not fail to identify any maternal deaths. Owing to our inability to cross-check cases with laboratory sources, our estimates of incidence should be regarded as minimum estimates.

### Interpretation

Our study found the incidence of severe maternal GBS sepsis to be substantially lower than that reported by a population surveillance study (1999–2005) in the USA (12 per 100 000, yearly range 11–14 per 100 000 live births)^22^ that had analysed all cases of confirmed invasive maternal GBS disease (with positive culture from a sterile body site). In this US study, 61% of women with invasive GBS disease had a miscarriage or stillborn infant, 30% had infants without apparent illness, 5% had live-born infants who developed clinical infections and 4% had termination of pregnancy. Similarly, a Canadian population-based surveillance study in 1996 investigating positive laboratory cultures of GBS from sterile body sites found the incidence of maternal GBS infection to be 41 per 100 000 total births, with only 73% of maternities resulting in the delivery of a live infant;^23^ interestingly, no live births were found to be infected with GBS. The estimate of incidence of severe maternal sepsis identified in the main study^14^ was compatible with estimates from other countries, suggesting that there was not significant under-reporting of cases.

The difference in the incidence of severe GBS sepsis found in the present study could be explained by the fact that only cases which met the criteria for severe sepsis were included in our case definition; however, in the light of our inability to cross-check data with laboratory sources, we cannot exclude the possibility of underascertainment. As our case definition was of ‘severe sepsis’, less severe forms of invasive maternal GBS disease would not have been included in our analysis. This is in contrast to other studies, including those noted above, which have measured the incidence of maternal GBS disease where the only inclusion criterion has been the presence of laboratory evidence of GBS from a sterile maternal body site, irrespective of the woman's clinical condition. Women included in these other studies may have an infection, but not necessarily severe sepsis. The difference in incidence observed between these studies using only laboratory sources and our study using clinical sources may, therefore, partly be explained by the fact that some cases of maternal GBS disease do not meet our definition of severe maternal sepsis. Our study also found infant morbidity and mortality associated with severe maternal GBS sepsis to be lower than other studies. A possible explanation could be the lack of correlation between severity of maternal and infant GBS disease. It is important to note, however, that 21% of infants of mothers with severe GBS sepsis developed sepsis; the raised odds of infant infection we observed is in line with that associated with maternal GBS carriage in other studies.^24^

It is, however, important to note that vaginal colonisation by GBS occurs in up to 20% of the population. Thus, some of the cases of severe sepsis which we found to have occurred in the presence of GBS-positive laboratory cultures that are not from sterile sites may have been caused by other pathogens with GBS being a coloniser and thus, these represent false-positive cases. This may partly account for the apparently better infant outcomes than in other studies.

These data help to inform the ongoing debate about antenatal GBS screening by providing information about the maternal burden of severe GBS sepsis in the UK. The data from this study, including the associated hospital and intensive care unit stay data and associated costs for both mothers and infants after diagnosis of sepsis, could contribute to a cost-effectiveness study of antenatal screening for GBS. Antenatal screening, which is now common practice in the USA and several European countries, aims to identify maternal GBS colonisation at 36–38 weeks gestation, through vaginal-rectal swabs, for management with intrapartum antibiotic prophylaxis (IAP). The UK is currently using a risk factor-based approach where IAP is only administered to women with antenatal or intrapartum risk factors for sepsis. Review of the latest available evidence has identified major limitations of antenatal GBS culture screening whereby the positive and negative predictive values of the test were found to be 50.5% and 91.7%, respectively.^4^ Real time PCR at the time of labour appears to be a more accurate way of testing colonisation at the time of labour, but evidence from studies assessing the validity of this method is still lacking.^4^ It is also important to note, as part of the assessment of the risks and benefits of screening and prophylaxis, that intrapartum prophylaxis with penicillin may have additional benefits for the mother in terms of protection against other invasive organisms, such as group A *Streptococcus*, an important cause of severe maternal sepsis identified in the original study.^14^

Vaccination of pregnant mothers against GBS provides another avenue for the prevention of maternal and neonatal GBS disease. Both laboratory research and early phase randomised controlled trials are currently ongoing investigating the immunogenicity and safety of potential GBS vaccines in pregnant women.^4^

## Conclusions

Confirmed severe maternal GBS sepsis appears to be rare, and these data can be taken into account when considering the risks and benefits of population screening and immunisation programmes. The rarity of the condition severely limits the power of this analysis to identify potential risk factors. A multinational study, such as through the International Network of Obstetric Survey Systems (INOSS),^25^ would be required to identify a larger numbers of cases for a robust investigation of associated factors. This study also raises questions concerning the association between the severity of maternal and infant disease that would also benefit from further investigation in a larger study.
